# The development of chimeric antigen receptor T-cells against CD70 for renal cell carcinoma treatment

**DOI:** 10.1186/s12967-024-05101-1

**Published:** 2024-04-18

**Authors:** Qinghui Xiong, Haiying Wang, Qiushuang Shen, Yan Wang, Xiujie Yuan, Guangyao Lin, Pengfei Jiang

**Affiliations:** Shanghai HRAIN Biotechnology Co., Ltd., 1238 Zhangjiang Road, Shanghai, 201203 China

**Keywords:** CD70, Chimeric Antigen Receptor T-Cells, Renal Cell Carcinoma, Nanobody

## Abstract

In this study, we investigated CD70 as a promising target for renal cell carcinoma (RCC) therapy and developed a potent chimeric antigen receptor T (CAR-T) cells for potential clinical testing. CD70, found to be highly expressed in RCC tumors, was associated with decreased survival. We generated CAR-T cells expressing VHH sequence of various novel nanobodies from immunized alpaca and a single-chain variable fragment (scFv) derived from human antibody (41D12). In our in vitro experiments, anti-CD70 CAR-T cells effectively eliminated CD70-positive tumor cells while sparing CD70-negative cells. The nanobody-based CAR-T cells demonstrated significantly higher production of cytokines such as IL-2, IFN-γ and TNF-ɑ during co-culture, indicating their potential for enhanced functionality. In xenograft mouse model, these CAR-T cells exhibited remarkable anti-tumor activity, leading to the eradication of RCC tumor cells. Importantly, human T cell expansion after infusion was significantly higher in the VHH groups compared to the scFv CAR-T group. Upon re-challenging mice with RCC tumor cells, the VHH CAR-T treated group remained tumor-free, suggesting a robust and long-lasting anti-tumor response. These findings provide strong support for the potential of nanobody-based CD70 CAR-T cells as a promising therapeutic option for RCC. This warrants further development and consideration for future clinical trials and applications.

**Supplementary Information:**

The online version contains supplementary material available at 10.1186/s12967-024-05101-1.

Renal cell carcinoma (RCC) is the most common form of kidney cancer and ranks as the fourteenth most prevalent cancer worldwide. In 2020, more than 400,000 new RCC cases were diagnosed, resulting in over 179,000 deaths [1]. Advanced RCC often shows resistance to chemotherapy, radiotherapy, and immunotherapy. Currently, the FDA has approved various treatments, including receptor tyrosine kinase inhibitors (RTKis) like VEGF/VEGFR inhibitors, mTOR inhibitors, immune checkpoint inhibitors (ICIs), and cytokines such as interferon-alpha (IFN-α) and interleukin-2 (IL-2). In clinical practice, first-line treatments for RCC often involve combining ICIs with RTKis or using a combination of two ICIs. However, for patients who do not respond to or relapse after these therapies, the limited treatment options highlight the need for innovative and mechanistically distinct treatments.

CD70, as a ligand of CD27, is a surface-expressed member of the tumor necrosis factor receptor superfamily [2]. CD70 expression is primarily found in a small subset of lymphoid lineage cells, including highly activated B and T cells, mature dendritic cells, and natural killer cells [3]. It is rarely expressed in normal non-hematopoietic tissues, and even in lymphoid tissues like the thymus, spleen, and lymph nodes, CD70 + cells are infrequent. Interestingly, CD70 is frequently expressed on T- and B-cell lymphomas and a significant fraction of solid tumors, such as thymic carcinoma, glioblastoma, renal cell carcinoma, osteosarcoma, and nasopharyngeal carcinoma [4–8]. Growing evidence suggests that tumor-associated CD70 expression may have immune suppressive properties [9, 10]. Therefore, there are ongoing efforts to target CD70 for therapeutic purposes, utilizing approaches like antibodies and cytotoxic antibody conjugates. Some of these candidates have entered clinical trials, such as cusatuzumab [11–14].

Chimeric antigen receptor T (CAR-T) cell immunotherapy has revolutionized cancer treatment by reprogramming a patient’s T cells to target and eliminate cancer cells. While CAR-T cell therapy has been highly effective against hematological malignancies [15], there is substantial evidence from numerous clinical trials indicating the need for further research to improve its efficacy against solid tumors [16–19].

Recent studies have shown that CAR-T cells targeting CD70 have displayed remarkable efficacy in combating a variety of tumors, including renal cell carcinoma, acute myeloid leukemia, glioblastoma, gliomas, head and neck squamous cell carcinoma, CD19-negative B-cell lymphoma, and melanoma, both in animal models and pre-clinical study [20–28]. Traditional CAR designs often use the single-chain variable fragment (scFv) as the antigen recognition element, which can sometimes trigger an immune response. The use of nanobodies (Nbs) can help mitigate this immunogenicity [29]. CAR-T cells engineered with Nbs as targeting elements have proven to be an effective strategy to prevent tumor immune evasion. These Nb-based CAR-T cells leverage the full potential of T cell immunotherapy, including tumor infiltration, cytokine release, and cytotoxic activity [30, 31].

In our present study, we developed CD70 CAR-T cells based on the VHH sequence of nanobodies as a novel therapeutic approach for RCC. We analyzed CD70 mRNA expression in RCC using The Cancer Genome Atlas (TCGA) database and confirmed the CD70 expression pattern in an RCC tissue array. Additionally, we identified various nanobodies from an antibody library through immunization in alpaca. This led to the design of a panel of CD70 CAR-T cells based on VHH sequences of nanobody, one of which demonstrated superior antitumor efficacy both in vitro and in vivo. These findings support CD70 as a potential target for immunotherapy, as well as early phase clinical testing of our novel CD70 CAR-T cells in patients with CD70-positive RCC.

## Materials and methods

### Cell lines

A-498 (HTB-44), 786-O (CRL-1932), ACHN (CRL-1611) and 769-P (CRL-1933) cell lines were purchased from ATCC and transduced to stably express firefly luciferase (Fluc) along with eGFP. ACHN-KO cell is the CD70 knock-out cell by CRISPR/Cas9. K562s (CRL-3343) cells were purchased from ATCC and transduced to stably express the CD70. All cell lines were tested for mycoplasma using the MycoAlert PLUS Mycoplasma Detection Kit (Lonza, Basel, Switzerland) and were negative.

### TCGA data analysis

The University of Alabama at Birmingham caner data analysis Portal (https://ualcan.path.uab.edu/index.html) was used to analysis CD70 expression in RCC patients based on the TCGA database. The survival analyses used the pathology data of CD70 on The Human Protein ATLAS (https://www.proteinatlas.org/).

### IHC (immunohistochemistry) staining

A tissue microarray (OUTDO Biotech, Shanghai, China) collected tumor tissue from patients diagnosed with RCC. Tissues were fixed in formaldehyde, decalcified, and embedded in paraffin. Rabbit polyclonal to CD70 (ab175389; Abcam, Cambridge, British) was used as the primary antibody. Staining was performed with the DAB staining kit (Abcarta, Suzhou, China). The primary CD70 antibody was diluted 1:800 and incubated at 4 °C overnight. The primary CD70 antibody was diluted to a ratio of 1:800 and incubated at 4 °C overnight. The staining results for all samples were assessed using the H-score, a semi-quantitative method that evaluates both staining intensity and the percentage of positive cells. H-scores were automatically calculated by Servicebio (Wuhan, China) with Aipathwell, a digital pathology image analysis software based on artificial intelligence (AI) learning.

### Plasmid construction

To generate a panel of CD70 VHH CAR constructs, cDNA of VHH chains derived from nanobody we had screened was commercially synthesized (AZENTA, Suzhou, China) and fused to CD8 hinge and transmembrane domain, and intracellular domains derived from human 4-1BB and CD3ζ which was synthesis based on genebank database and cloned into the 3rd generation retroviral vector MSGV1 using In-fusion cloning (Takara, Otsu, Japan). A CD70 scFv CAR was generated by linking the VH and VL chains derived from a published antibody 41D12 [32].

### Retroviral production

Retroviruses were generated by first transfecting 70% confluent HEK 293vec-RD114 with transfer plasmid that was complexed with PEI at a DNA: PEI mass ratio of 1:3. For a confluent T25 flask, 3 μg of transfer plasmid was used for transfection. Media was changed 18 h after transfection and retroviral particles were harvested in the supernatant 48 and 72 h after transfection. The supernatant was then filtered through a 0.45 μm low protein binding filter and stored at − 80 °C.

### Human T cell activation, transduction and expansion

Cryopreserved PBMC was thawed at a 37 °C water bath, washed and suspended in CAR-T culturing medium X-VIVO15 (LONZA, Valais, Switzerland) contented 1% GlutaMAX (ThermoFisher Scientific, Waltham, MA), 1% HEPES (ThermoFisher Scientific, Waltham, MA), 0.2% N-Acetyl-l-cysteine and 5% human plasma. After rested for 2 h at 37 °C with 5% CO_2_, PBMC has been washed and suspended with DPBS (Corning Incorporated, Corning, NY) for CD3 + T cell positive selection. CD3 + T cells was made by co-incubating CTS Dynabeads CD3/CD28 (ThermoFisher Scientific, Waltham, MA) with PBMC at a 1:1 ratio at room temperature followed by magnetic capture of bead-bound cells on DynaMag-5 Magnet (ThermoFisher Scientific, Waltham, MA). Selected cells were suspended in CAR-T culturing medium supplemented with 300 IU/mL IL-2 (SL Pharm, Beijing, China) and incubated at 37 °C with 5% CO_2_ for 24 ± 4 h to complete T cell activation. After which T lymphocytes were transduced on Retronectin-coated plates (Takara, Otsu, Japan) and then expanded in CAR-T culturing medium supplemented with IL-2 for 4 days prior to characterization and maintained at a density of 3 × 10^5^–2 × 10^6^ cells/ml.

### Flow cytometry

Cells were washed with 1xPBS (Sigma, Louis, MO), then surface stained by incubating with antibodies for 30 min at 2 ~ 8 °C. They were subsequently washed again prior to flow analysis on Sony SA3800. Anti-CD4 (clone OKT4), anti-CD8a (clone RPA-T8), anti-PD-1 (clone EH12.2H7), anti-TIM3 (clone F38-2E2), anti-LAG3 (clone 7H2C65), anti-CD3 (clone UCHT1 or clone HIT3a), anti-CD62L (clone DREG-56), anti-CD45RO (clone UCHL1), anti-CD69 (clone FN50), anti-CD137 (clone 5F4), anti-CD25 antibodies (clone BC96), and Streptavidin were purchased from Biolegend (San Diego, CA). After collecting the supernatant, protein purification was accomplished with a protein-A affinity Beads. Biotin were labeled to purified CD70 protein with kit.

### Cytotoxicity assay

CD70 positive tumor cells 786-O, A498, 786-P, ACHN and K562-CD70 expressing firefly luciferase (Fluc) were co-cultured with CD70 CAR-T cells for 18 h in IL-2 deficient media at various E: T ratios. Cells were lysed and added of luciferin substrate from the ONE-Glo Luciferase Assay System (Promega, Madison, WI). The resulting luminescent signal was measured using a Tecan Infinite M200 Pro. Signals were normalized to negative controls containing only target cells. Cells were tested with the ONE-Glo Luciferase Assay System after co-cultured for 48, 72, 96, and 120 h. The co-culture well is replenished with fresh medium every 2–3 days. For serial killing assays, target cells and CAR-T cells were co-cultured as described above at E: T ratios of 1:1. Every two- or three-days target cells were added into the co-cultured system. Target cell viability in the spent plate was read out by ONE-Glo reagent.

### Cytokine release assay

Collected the supernatant during cytotoxicity assays to measure released cytokines using Cytometric Bead Array (CBA) Human Th1/Th2/Th17 Cytokine Kit (BD, Franklin Lakes, NJ). The data were analyzed using Flowjo10.8.1 software.

### Xenograft model and in vivo tests

All animal studies were conducted with the approval of the BioDuro-Sundia Institutional Animal Care and Use Committee (IACUC), and animal welfare and use adhered to the guidelines set forth by the Association for the Assessment and Accreditation of Laboratory Animal Care International (AAALAC). Female NOG mice (NOD.Cg-PrkdcscidIL2rgtm1Sug/JicCrl, Vital River, Beijing, China) of 5–7 weeks of age were maintained in specific-pathogen-free conditions, with a daily cycle of 12 h of light and 12 h of darkness. Continuous health monitoring was performed regularly. Mice were humanely euthanized when they exhibited symptoms of clinically overt disease, such as decreased feeding, reduced activity, abnormal grooming behavior, or a hunched back posture, or when they experienced excessive weight loss amounting to 20% of their body weight.

In these experiments, each animal received an injection of 5 × 10^6^ ACHN cells. Once the average tumor size reached approximately 100 mm^3^, frozen stock solution, non-transduced T cells (NT), or CAR-positive T cells were intravenously administered via the tail vein. In tumor re-challenge experiments, tumor-bearing mice received a subcutaneous injection of 5 × 10^6^ ACHN cells into the right flank. Tumor volume and body weight were measured and recorded twice weekly.

Blood samples (50–100 μL per mouse) were collected from mice weekly to measure T cell frequency and absolute numbers. Plasma was separated by centrifugation to measure cytokine levels using the Human Th1/Th2/Th17 CBA Kit. Mice were euthanized if they exhibited signs of illness or when the tumor burden reached 1500 mm^3^.

### Statistical analysis

The results presented in this study represent data obtained from a minimum of three independent experiments. Statistical analyses were conducted using GraphPad Prism 8.0. Two-tailed Student’s t-test and two-way ANOVA were employed for statistical analysis, with statistical significance defined as P < 0.05. The number of asterisks indicates the significance level: * for 0.05, ** for 0.01, *** for 0.001,**** for 0.0001 in bar graph.

## Results

### CD70 expression in RCC

**Fig. 1:**
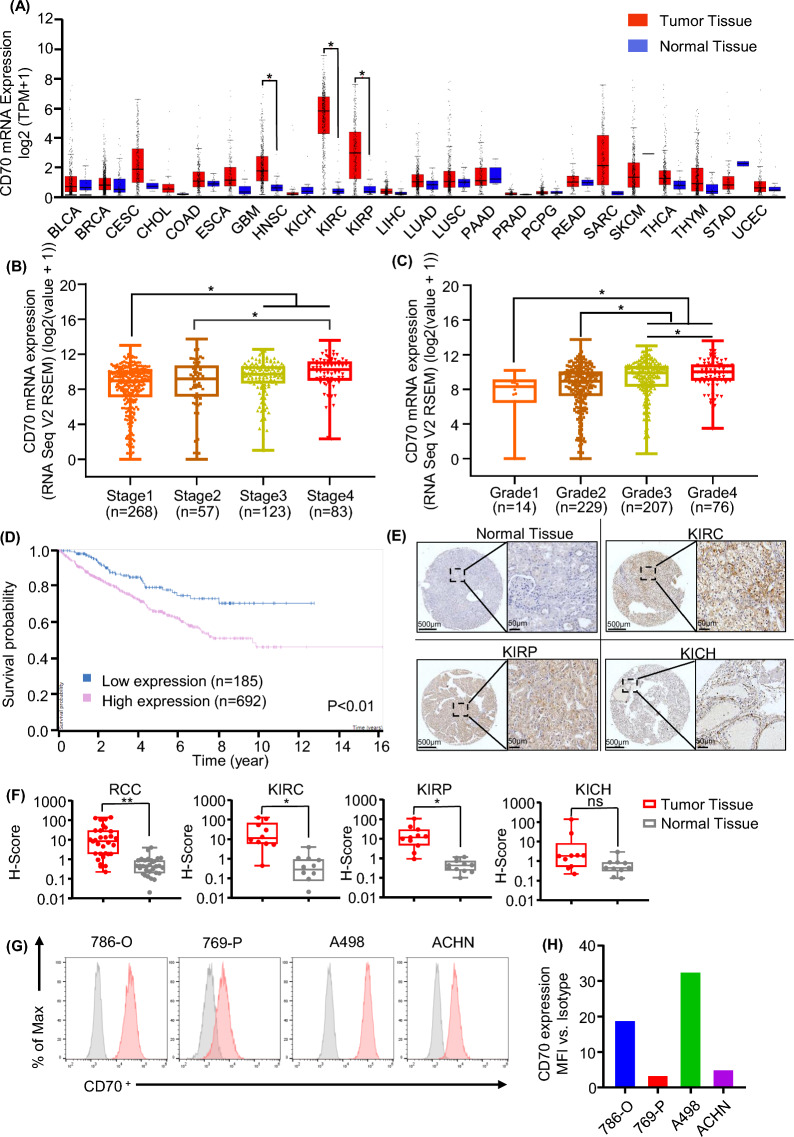
CD70 Expression Analysis. **A** mRNA level of CD70 in TCGA sequencing database demonstrated various malignant tissue express higher CD70 compared with normal tissue, especially in KIRC and KIRP, two major types of RCC **B** CD70 mRNA level in RCC samples according to patient’s clinical stages showed significantly higher expression CD70 associated with later stages (Stage 3 and Stage 4) (n = 72 normal, n = 267 stage 1, n = 57 stage 2, n = 123 stage 3, n = 4 stage 4). **C** CD70 mRNA level in RCC samples according to pathological grading, showed higher CD70 expression related with higher grades (Grade 3 and Grade 4) (n = 72 normal, n = 14 grade 1, n = 229 grade 2, n = 206 grade 3, n = 76 grade 4). *P < 0.05, **P < 0.01 ***P < 0.001, ****P < 0.0001 by unpaired Student *t* test. **D** Lower RCC patients survive related with higher CD70 expression (n = 185 low expression, n = 692 high expression) in TCGA database. **E** Representative immunohistochemical staining images for CD70 expression in samples from patients with KIRC, KIRP and KICH (Kidney Chromophobe). **F** Comparison of CD70 expression detected by IHC in samples from 109 RCC patients and 6 normal tissues. Total RCC patients and KIRC, KIRP but not KICH subtypes patients expressed significantly higher CD70 than the normal tissues. *P < 0.05, **P < 0.01 by unpaired Student *t* test. **G** Cell surface CD70 expression in RCC cell lines detected by flow cytometry. All RCC cell lines (786-O, 769-P, A498, ACHN) expressed CD70 on cell surface. **H** Measurement of cell surface CD70 expression level in RCC cell line. Median fluorescence intensity (MFI) ratio calculated by the MFI detected with CD70 antibody divided by antibody isotypes control.

In our evaluation of CD70 as a potential cell surface target for RCC immunotherapy, we conducted an analysis of CD70 mRNA expression across the TCGA database by pan-cancer view. For all 24 types of cancer, kidney clear cell carcinoma (KIRC) and kidney papillary cell carcinoma (KIRP), the two major subtypes that accounts for over 85% of RCC in adults, have highest expression level compared with normal tissues (Fig. 1A). The analysis of 533 KIRC samples revealed that the higher CD70 expression always related higher grade and late cancer stage (Fig. 1B, C). By the pathology atlas of survival analysis, CD70 is a prognostic marker and high expression is unfavorable in renal cancer. For total 877 RCC patients recorded in TCGA database, 185 patients have lower CD70 mRNA level have significantly longer survive time than 692 patients with higher CD70 expression (Fig. 1D). To investigate CD70 protein level in RCC sample, we detected CD70 expression by IHC. The two major subtypes (KIRC and KIRP) of RCC samples showed significantly higher signal than the normal kidney tissue (Fig. 1E, F). CD70 was also detectable on the cell surface in all tested RCC cell lines (Fig. 1G, H). These data strongly indicated that CD70 is a promising cancer therapy target.

### Generation of CD70 CAR-T cells

**Fig. 2:**
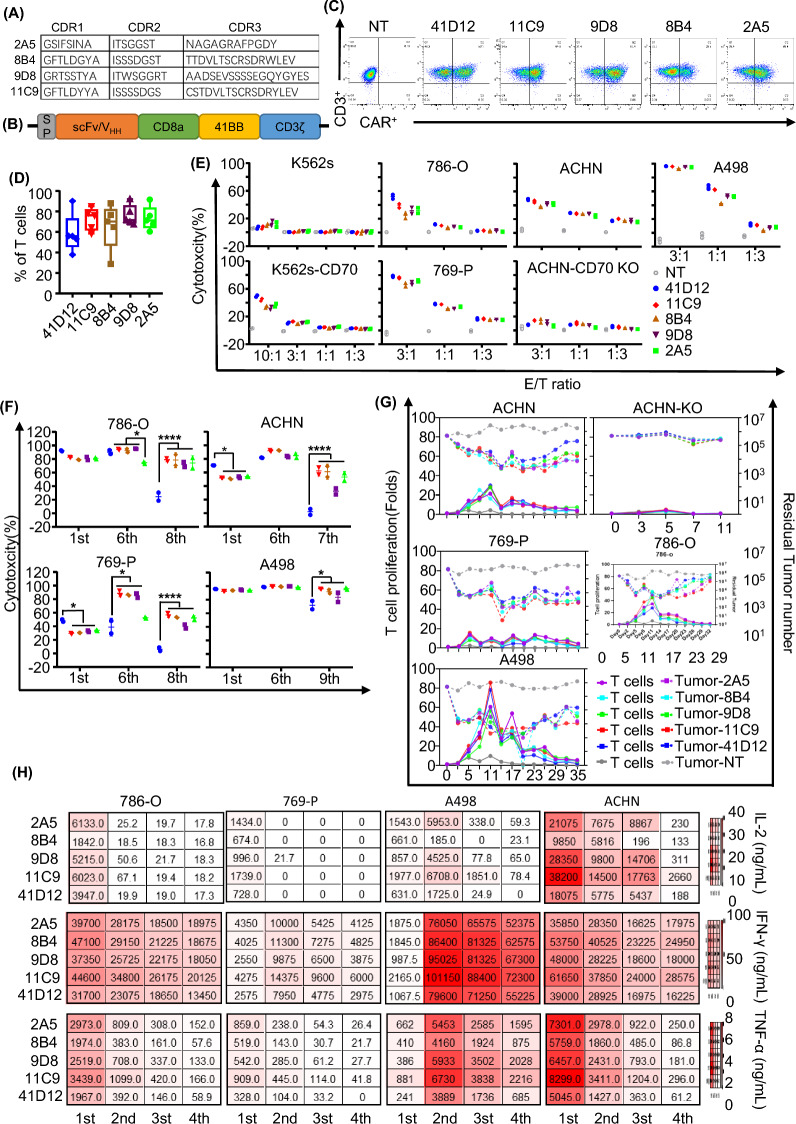
Generation and Characterization of CD70 CAR-T Cells **A** The amino acid sequences of CDR1-3 region of novel nanobody isolated from the phage library and identified as the specific binder to CD70. **B** CAR construct with CD8*α* signal peptide (SP), scFv (41D12) or VHH (Nanobody), CD8*α* hinge and transmembrane domain, intracellular 4-1BB co-stimulatory domain, and intracellular CD3*ζ* activation domain. **C** CD70 CAR expression levels of 5 different donors measured by flow cytometry after transduction of activated T cells. CAR detected by biotin-labeled CD70 protein and BV421-conjugated streptavidin. **D** The percentage of CD70 CAR expressing T cells from 5 different health donors. All CAR highly expressed on T cell surface. **E** Antigen specific cytotoxicity of CAR-T cells. As CD70 positive cell lines (786-O, 769-P, ACHN, A498, K562 transduced with CD70) and CD70-negative cell lines (K562-WT, ACHN-CD70 KO) as a control, assessed by luciferase assay. All CAR-T cells were potent to kill CD70 positive target cell without cytotoxicity to antigen negative cells. **F** Antigen specific cytotoxicity of CD70 CAR-T cells against CD70-positive RCC cell lines (786-O, 769-P, ACHN, A498) during serial killing assays using luciferase assays. *P < 0.05, ****P < 0.0001 by two-way ANOVA with Dunnett's multiple comparisons test adjusted p value. **G** Fold change of CD3 + T cells in CD70 CAR-T co-culture with RCC cell lines during antigen stimulation in repetitive co-culture assay. **H** Analysis of cytokine production in the supernatants from repetitive co-cultures of RCC cells and CAR-T cells. The heatmap showed an increase in cytokines, including IL-2, TNF-α, IFN-γ, in the CD70 CAR-T cell groups

Alpaca immunized with home-made CD70 extracellular domain (acid 39–193), antibody phage library constructed, after biopanning, we got the CD70 binder. The affinity of new nanobody against CD70 measured by BLI assay and kinetic analysis. The result published in other place [33]. Our study generated a panel of CD70 VHH-based CAR constructs from 4 novel nanobodies (Fig. 2A) and a scFv-based CAR constructs from human 41D12 (Fig. 2B). CAR-T cells were generated by retroviral transduction, with the median percentage of CAR-positive T cells ranging between 55.9 and 76.3%, as the expression of CAR is not detectable in non-transduced T cells (NT) (Fig. 2C, D). In downstream experiments, we adjusted the number of T cells according to the percentage of CAR-positive cells. Further phenotype analysis revealed that anti-CD70 CAR-T cells induced T-cell activation and exhausting, as measured by a decreased frequency of T naïve-like (CD45RO-, CD62L +), and an increased CD25 + T cells in comparison with NT cells; exhausted biomarker TIM-3 was also upregulated in anti-CD70 CAR-T cells (Additional file 1: Fig. S1A–C). CD8 + composition was lower in CD70-VHH CAR-T cells in comparison with NT cells but did not show significant difference among different CAR constructs (Additional file 1: Fig. S1D). CD70 expression was also measured in T cells of PBMC (Additional file 2: Fig. S2A). As the previous reported, CD70 is rare in inactivated T cells, and based on our result, there were more CD70 positive cells in CD4 positive population.

### In vitro antitumor activity

All CAR-T cells showed specific cytotoxicity against the CD70 positive kidney tumor cell lines (786-O, 769-P, A498, ACHN). The CD70-knockout variant of ACHN (ACHN-CD70 KO), the efficiency of CD70-knockout in which was confirmed by FACS (Additional file 2: Fig. S2B and C), showed resistance to CAR-T killing. K-562 s cell which is multipotential, hematopoietic malignant cell not origin or related kidney tissue, without CD70 expression (Additional file 2: Fig. S2B and C), also showed potency to resistant. But for CD70 overexpressed k562s cells (k562-CD70, CD70 expression showed in Additional file 2: Fig. S2B and C), CAR-T cells showed potent cytotoxicity. This killing pattern confirmed our CAR-T cell specifically recognize and eradicated CD70 positive cells. There was no significantly different cytolytic activity among all CAR constructs (Fig. 2E). Because CAR-T-cell efficacy is determined not only by its cytolytic activity, but also by the capacity of antigen-specific proliferation upon tumor challenge, we performed repetitive co-culture assays, in which CD70 CAR-T cells were repeatedly challenged with CD70-positive cells and ACHN-CD70 KO cells. All CD70 CAR-T eliminated tumor cells and proliferated during the first round coculture, but lost their capacity of killing and/or proliferation during repetitive co-cultures. All CD70 VHH CAR-T cells eliminated tumor cells for at least 7 consecutive cocultures. However, 41D12 CAR-T cell undetectable during the co-cultures much sooner than other CAR-T cells (Fig. 2F). The antitumor activity of CD70 CAR-T cells was consistent with the ability of expansion after tumor cell challenge (Fig. 2G). We also measured cytokine production by CD70 CAR-T cells during the first 4 rounds repetitive stimulation. 11C9 CAR-T cells produced the highest levels of Th1/Tc1cytokines, such as IL-2, IFN-γ and TNF-α (Fig. 2H).

### In vivo efficacy against RCC

**Fig. 3:**
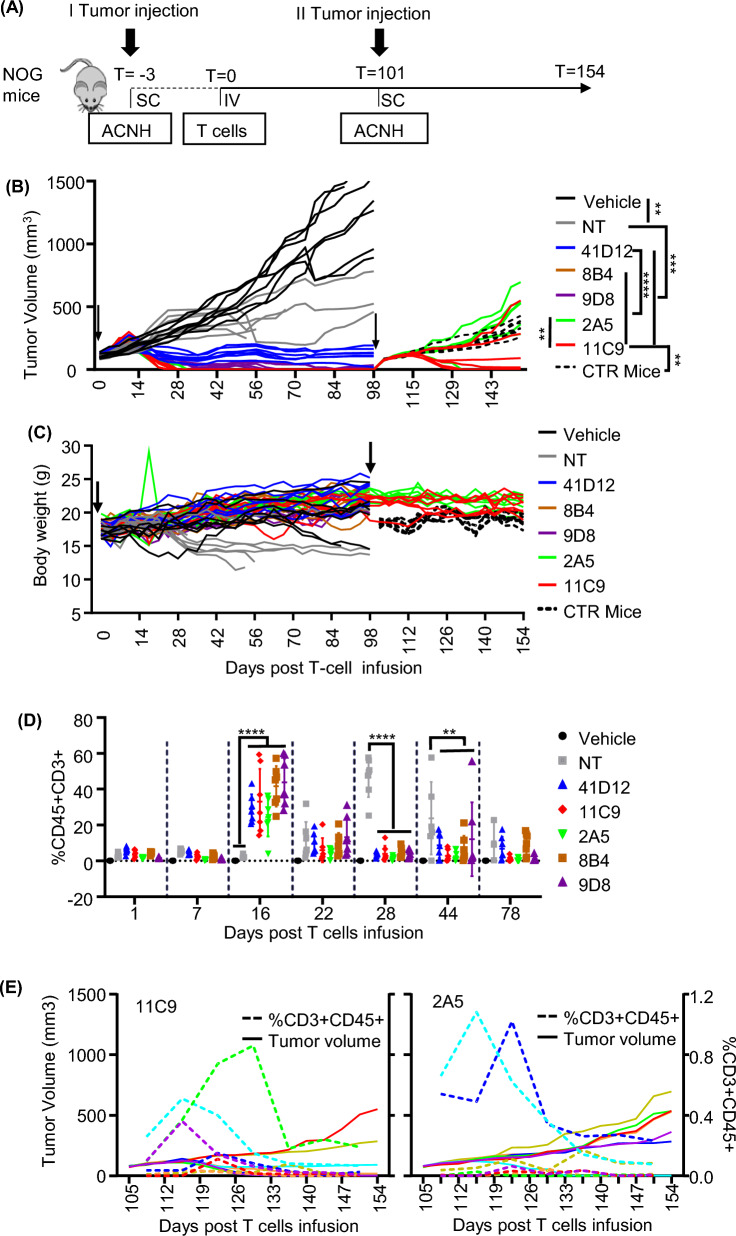
In Vivo Antitumor Activity of CD70 CAR-T Cells. 11C9 and 2A5 CAR-T cells treatment showed higher potency to eliminate tumor cell. **A** Schematic representation of the ACHN xenograft RCC model in NOG mice. Mice with complete responses after CAR-T cell treated group were re-challenged on day 101 with ACHN cells by S.C. to the flank of other side. **B** Tumor growth curve and **C** bodyweight changes of mice treated with CD70 CAR-T, non-transduced (NT), or vehicle control (Cell Cryopreservation Solution). Tumor volumes were compared by Student’s *t* test, **P < 0.01, ***P < 0.001, ****P < 0.0001. **D** The percentage of human CD3 + CD45 + cells in peripheral blood of mice detected by flow cytometry after CAR-T treatment. **P < 0.01, ****P < 0.0001 by Student’s *t* test. **E** Tumor volume after ACHN re-challenge and the percentage of human CD3 + CD45 + cells in individual mice of the 11C9 and 2A5 CAR-T treated group. After re-challenge, growing tumor cell in the other side of flank boosted CAR-T cell proliferation, then at least in part of mice of 11C9 treated mice, the tumor cell eradicated again

In mouse xenograft model, CD70 CAR-T cells efficiently controlled RCC tumor growth, leading to complete remission. Our model used NOG mice received subcutaneous injections of ACHN cells. When the average tumor size was about 100 mm^3^, animals received a single IV dose of 2 × 10^7^ CD70-CAR or non-transduced T cells as control. Tumor growth curve showed VHH CD70 CAR-T cells efficiently controlled RCC growth, leading to complete remission in mice by day 28 (11C9 and 8B4 CAR-T), day 31 (2A5 CAR-T) and 5/7 mice by day 66 (9D8 CAR-T). In contrast, no mice received 41D12 CAR-T cells achieved complete remission (Fig. 3A). CD70 CAR-T cell infusion didn’t affect mice bodyweight, but NT T cell treated mice lost bodyweight after day 21 post T cells infusion which might be attribute to Graft versus Host Disease (GvHD) effect induced by unspecific expansion of NT cells (Fig. 3C).

**Fig. 4:**
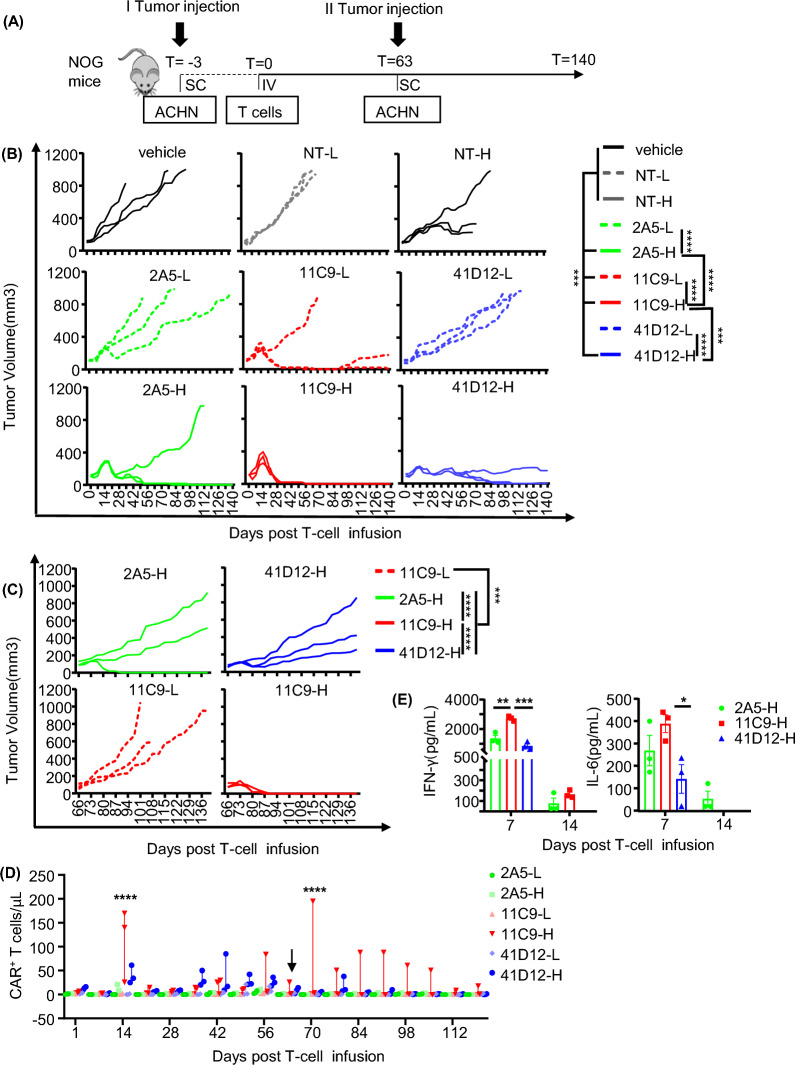
Comparison of In Vivo Antitumor Activity of VHH CD70 CAR-T. 11C9 CAR-T cells showed highest eradication potency against tumor cell. **A** Schematic representation of the ACHN xenograft RCC model in NOG mice. These mice were inoculated with 5 × 10^6^ ACHN cells subcutaneously and received 2 dosages of CAR-T cells (1 × 10^6^ or 5 × 10^6^ CAR + cells/mouse) intravenously. All mice in the 5 × 10^6^ CAR + dose CAR-T treated groups (2A5-H,11C-H,41D12-H) and 11C9 1 × 10^6^ CAR + treated groups (11C9-L) were re-challenged on day 63 with 5 × 10^6^ ACHN cells. **B** Tumor growth curves after the first tumor injection in all mice treated with CD70 CAR-T, non-transduced (NT), or vehicle control (Cell Cryopreservation Solution). **C** Tumor growth curves after re-challenge. Only 11C9-H mice showed robust tumor growth inhibition. **D** Human T cell counts in peripheral blood of mice measured by flow cytometry. ***P < 0.001, ****P < 0.0001 by Student’s *t* test. **E** The concentration of human IFN-γ and IL-6 in peripheral blood of mice at day7 and day 14 after T cell infusion were analyzed by CBA. *P < 0.05,**P < 0.01, *** P < 0.001 by Student’s t test

We compared the antitumor activity of 2A5, 11C9 and 41D12 CAR-T cells in vivo in another animal experiment (Fig. 4A). ACHN loaded NOG mice received a single IV dose of 5 × 10^6^ or 1 × 10^6^ CD70 CAR-T cells. 11C9 CAR-T cells still efficiently controlled RCC growth and leading to complete remission in all mice by day 28 (5X10^6^ dose group) and 2/3 mice by day 35 (1X10^6^ dose group). In contrast, 2 mice achieved complete remission in high dose group of 2A5 (by day 52) and 41D12 CAR-T (by day 84), and no one in low dose group achieved completely remission (Fig. 4B).

### Long-term memory

In cured mice, re-challenging with tumor cells led to long-term tumor control, indicating the establishment of immunological memory.

In our first In Vivo study, human T cell (CD3 + CD45 +) cells in peripheral blood achieved peak at day 16, after that it deceased dramatically. Since day 28, the presence of human CD3 + CD45 + T cells in CAR-T treated mice was very low and close to the detection limitation. To evaluated capability of persistence against tumor, mice were re-challenged subcutaneously inoculated with ACHN cells at day 101. It showed rapid progression of the tumor in control (CTR mice) and 2A5 treated mice. 11C9 CAR-T showed a long-term antitumor activity, rechallenged tumor growth comparable with control in the first 2 weeks. After that, 2 mice tumor still growing, other 5 mice showed retarded tumor growth. To the end of experiments, 4 mice were tumor-free or tumor too small to measure, 1 mouse tumor volume decreased to 72 mm^3^ and keep the size about 1 month (Fig. 3B).

In our second in vivo test, mice treated much lower dose of CAR-T compared with our first test, were re-challenged with ACHN cells at day 66 and followed up for another 70 days. 11C9 CAR-T still showed a long-term antitumor activity after 5X10^6^ CAR-T treated, all mice maintained tumor-free; the 2A5 and 41D12 CAR-T treated group also retarded tumor growth after rechallenge, but just partially or none mice achieved CR (Fig. 4C).

### CAR-T cell expansion

We investigated CAR-T-cell expansion and persistence in vivo. In the first in vivo test, we analyzed the percentage of circulating CD45 + CD3 + human cells over time in all mice and observed significantly greater expansion in day 16 after T cell infusion in mice treated with VHH CAR-T cells than that of 41D12 CAR-T (Fig. 3D). After ACHN re-challenged, T cells expanded again in all mice (Fig. 3E).

In the second in vivo test to confirm the anti-tumor ability of 2A5, 11C9 and 41D12 CAR-T cells, we measured the absolute number of circulating CD45 + CD3 + human cells every week post cells infusion, 11C9 CAR-T cells induced highest T cells expansion than the other CD70 CAR-T cells at the first expansive peak in day 14, and in day 70 (day 7 after ACHN re-challenge), there was an obvious expansion of T cells observed (Fig. 4D).

We also evaluated the concentration of human Th1/Th2/Th17 cytokines (IL-2, IL-4, IL-6, IL-10, IL-17A, IFN-γ, and TNF-α) in the peripheral blood of mice on days 7 and 14 post T cell infusion (Fig. 4E). None of these cytokines were detectable in the vehicle and NT groups. In the high-dose CD70 CAR-T infusion group, only IFN-γ and IL-6 were detectable.When comparing the three CD70 CAR-T infusion groups (2A5, 41D12, and 11C9), IFN-γ plasma levels were highest in the 11C9 group on Day 7, consistent with the in vitro test results. However, by Day 14, IFN-γ levels had sharply declined, and the difference between the 2A5 and 11C9 groups became insignificant, although IFN-γ levels remained higher in the 11C9 group and were undetectable in the 41D12 group.Regarding IL-6, levels were higher in the 11C9 group compared to the 41D12 group on Day 7, but not compared to the 2A5 group. However, by Day 14, IL-6 levels had dropped significantly, and it was undetectable in both the 11C9 and 41D12 groups.

## Discussion

In our current study, we have developed an innovative approach utilizing nanobody-based CD70 CAR-T cells, demonstrating remarkable anti-tumor activities both in vitro and in vivo. Notably, the CD70 CAR-T based on the VHH sequence of the novel nanobody 11C9 displayed superior anti-RCC activity in vivo. Mice treated with a single dose of CAR-T achieved complete remission by day 28 and maintained for over than 120 days at the end of study. Furthermore, without further treatment, these mice exhibited strong immune memory against RCC rechallenge.

According to previous research, certain immune factors such as IL-2, IL-7, IL-12, IL-15, IL-18, IL-21, IL-23, IFN-γ, and TNF-α have demonstrated significant effects in enhancing CAR-T therapy [34, 35]. For instance, IL-2 plays a crucial role in T cell proliferation and effector function, and the orthogonal IL-2 receptor/ligand system has been shown to maximize the efficacy of CAR T therapies by selectively expanding and activating CAR T cells in vivo.

In our in vitro studies, we observed higher levels of IL-2, IFN-γ, and TNF-α induction with 11C9 CAR-T compared to other variants. However, when we measured human Th1/Th2/Th17 cytokine levels in peripheral blood, we found that IFN-γ and IL-6 were highest in the 11C9 CAR-T group on day 7. Unfortunately, the concentration of IL-2 and TNF-α was too low to detect in the mouse model. This limitation highlights the challenge posed by the limited volume of mouse blood available for collection, which impedes the detection of cytokines.

We observed higher levels of cytokines induced and more significant expansion of 11C9 based CAR-T compared to others in our study. Several other factors, such as moderate affinity, critical epitope targeting and a higher CD4/CD8 ratio may also contribute to the anti-tumor activity, although further investigation is needed. Importantly, our CD70 CAR-T cells were successfully generated without succumbing to fratricide induced by the CD70 on the activated T cells. This avoidance of fratricide may be attributed to *cis* masking of CD70 by CAR expression [22]. Further studies are required to optimize the CAR-T manufacturing process.

CD70 represents a highly attractive therapeutic target in RCC treatment, with numerous academic and industry groups are dedicating to developing CD70 CAR-T products. Notably, Allogene Therapeutics has reported the success of CD70 scFv-based allogeneic CAR-T cells (ALLO-316) exhibited potent anti-tumor activity. ALLO-316 also demonstrated a favorable safety profile, with no significant adverse effects observed during the preclinical studies [22]. In addition, CRISPR Therapeutics has developed another allogeneic CD70 CAR T cells (CTX-130), which has shown early signs of clinical activity in advanced RCC patients, according to findings from the phase 1 COBALT-RCC trial (NCT04438083) [36]. Currently, both ALLO-316 and CTX-130 represent the rapidly advancing CD70 CAR-T candidates for RCC treatment in clinical development. However, it is essential to note that despite significant advancement in allogeneic CAR-T therapies, they still exhibit lower efficacy and raise more safety concern than the autologous counterparts.

Autologous CD19 CAR-T cell products have been the most successful CAR-T cell therapy and have led the FDA approvals of three such products: tisagenlecleucel (Kymriah, Novartis), axicabtagene ciloleucel (Yescarta, Kite Pharmaceuticals), and brexucabtagene autoleucel (Tecartus, Kite Pharmaceuticals) [37–40]. Autologous CAR T cells have shown robust anti-tumor activity against hematological malignancies targeting BCMA, CD20, CD22, and CD30 [41–44]. While autologous CD19-CAR T cells have clinical and economical limitations, including challenges associated with leukapheresis, manufacturing and efficacy in heavily pre-treated patient population, they have shown a consistent safety profile and durable responses [45]. On the other hand, allogeneic CAR-T cells pose their own set of challenges, such as the potential to induce graft-versus-host-disease and the risk of immune-mediated rejection by the host [46]. Importantly, the regulatory path and the Chemistry, Manufacturing, and Controls (CMC) requirements for autologous CAR-Ts are well established. Therefore, autologous CAR-T therapies have been shown to be a more mature and safer product. We firmly believe that autologous CD70 CAR-T cell therapies will address the unmet needs of RCC patients.

In conclusion, we have successfully developed a nanobody-derived CD70-specific autologous CAR-T cell therapy for RCC treatment. Based on this research, intensive preclinical studies and CMC research led to the approval from the Center for Drug Evaluation (CDE) of the National Medical Products Administration (NMPA) this year. Clinical trial of autologous CD70 CAR-T will conduct to address the more critical questions surrounding this novel RCC therapy.

### Supplementary Information

**Additional file 1: Figure S1.** The Characteristic of CD70 CAR T cells. (A) Phenotypical characterization of CD70 CAR T cells by flow cytometry. differentiated phenotype was determined by the expression of CD45RO and CD62L in 5 different donors. *P<05, by paired Student t test. (B) The activated biomarker expression of CD70 CAR T cells by flow cytometry. Activation was measured by the expression of CD69, CD25, CD137 in 5 different donors ***P<0.001, by paired Student t test. (C). The exhausted marker expression of CD70 CAR T cells by flow cytometry. exhaustion was measured by the expression of Tim3, PD-1 and Lag3 in 5 different donors *P<05, by paired Student t test. (D) The percentage of CD8+ cells in CD3+ cells of CD 70 CAR T cells by flow cytometry. *P<05; ns, not significant, by paired Student t test.

**Additional file 2: Figure S2.** CD70 expression of human tumor cell lines and T cells in PBMC (A) Analysis of CD70 expression in T cells of a healthy donor’s PBMC by flow cytometry. (B) Analysis of CD70 expression in ACHN wild-type, ACHN-CD70 knockout, k562s wild-type and k562s with CD70 overexpression by flow cytometry. (C)Median fluorescence intensity (MFI) quotients of CD70 versus the respective isotypes on tumor cell lines.

## Data Availability

The data and materials supporting the findings of this study are available within the paper and its Additional files information. Additional information may be obtained from the corresponding author upon reasonable request. This disclosure statement is intended to transparently communicate any potential influences or considerations related to our affiliation with Shanghai HRAIN Biotechnology Co., Ltd.. We affirm our commitment to maintaining the integrity and impartiality of the research presented in this paper.

## References

[1] Globocan: In https://gco.iarc.fr/today/home, edited by WHO. 2020.

[2] Hintzen RQ, Lens SM, Beckmann MP, Goodwin RG, Lynch D, van Lier RA (1994). Characterization of the human CD27 ligand, a novel member of the TNF gene family. J Immunol.

[3] Hintzen RQ, Lens SM, Lammers K, Kuiper H, Beckmann MP, van Lier RA (1995). Engagement of CD27 with its ligand CD70 provides a second signal for T cell activation. J Immunol.

[4] Hishima T, Fukayama M, Hayashi Y, Fujii T, Ooba T, Funata N, Koike M (2000). CD70 expression in thymic carcinoma. Am J Surg Pathol.

[5] Held-Feindt J, Mentlein R (2002). CD70/CD27 ligand, a member of the TNF family, is expressed in human brain tumors. Int J Cancer.

[6] Jilaveanu LB, Sznol J, Aziz SA, Duchen D, Kluger HM, Camp RL (2012). CD70 expression patterns in renal cell carcinoma. Hum Pathol.

[7] Pahl JH, Santos SJ, Kuijjer ML, Boerman GH, Sand LG, Szuhai K, Cleton-Jansen A, Egeler RM, Bovee JV, Schilham MW, Lankester AC (2015). Expression of the immune regulation antigen CD70 in osteosarcoma. Cancer Cell Int.

[8] Agathanggelou A, Niedobitek G, Chen R, Nicholls J, Yin W, Young LS (1995). Expression of immune regulatory molecules in Epstein-Barr virus-associated nasopharyngeal carcinomas with prominent lymphoid stroma. Evidence for a functional interaction between epithelial tumor cells and infiltrating lymphoid cells. Am J Pathol.

[9] Yang ZZ, Novak AJ, Ziesmer SC, Witzig TE, Ansell SM (2007). CD70+ non-Hodgkin lymphoma B cells induce Foxp3 expression and regulatory function in intratumoral CD4+CD25 T cells. Blood.

[10] Gong L, Luo J, Zhang Y, Yang Y, Li S, Fang X, Zhang B, Huang J, Chow LK, Chung D (2023). Nasopharyngeal carcinoma cells promote regulatory T cell development and suppressive activity via CD70-CD27 interaction. Nat Commun.

[11] Leupin N, Zinzani PL, Morschhauser F, Dalle S, Maerevoet M, Michot JM, Ribrag V, Offner F, Beylot-Barry M, Moins-Teisserenc H (2022). Cusatuzumab for treatment of CD70-positive relapsed or refractory cutaneous T-cell lymphoma. Cancer.

[12] De Meulenaere A, Vermassen T, Creytens D, De Keukeleire S, Delahaye T, Deron P, Duprez F, Fung S, Pauwels P, Ferdinande L, Rottey S (2021). An open-label, nonrandomized, phase Ib feasibility study of cusatuzumab in patients with nasopharyngeal carcinoma. Clin Transl Sci.

[13] Riether C, Pabst T, Hopner S, Bacher U, Hinterbrandner M, Banz Y, Muller R, Manz MG, Gharib WH, Francisco D (2020). Targeting CD70 with cusatuzumab eliminates acute myeloid leukemia stem cells in patients treated with hypomethylating agents. Nat Med.

[14] The CD70 Antibody Cusatuzumab Shows Promise in Acute Myeloid Leukemia. Cancer Discov 2020; 10(9): 1251.

[15] Zhang X, Zhu L, Zhang H, Chen S, Xiao Y (2022). CAR-T cell therapy in hematological malignancies: current opportunities and challenges. Front Immunol.

[16] Sterner RC, Sterner RM (2021). CAR-T cell therapy: current limitations and potential strategies. Blood Cancer J.

[17] Maalej KM, Merhi M, Inchakalody VP, Mestiri S, Alam M, Maccalli C, Cherif H, Uddin S, Steinhoff M, Marincola FM, Dermime S (2023). CAR-cell therapy in the era of solid tumor treatment: current challenges and emerging therapeutic advances. Mol Cancer.

[18] Marofi F, Motavalli R, Safonov VA, Thangavelu L, Yumashev AV, Alexander M, Shomali N, Chartrand MS, Pathak Y, Jarahian M (2021). CAR T cells in solid tumors: challenges and opportunities. Stem Cell Res Ther.

[19] Zhang ZZ, Wang T, Wang XF, Zhang YQ, Song SX, Ma CQ (2022). Improving the ability of CAR-T cells to hit solid tumors: challenges and strategies. Pharmacol Res.

[20] Zhu G, Zhang J, Zhang Q, Jin G, Su X, Liu S, Liu F (2022). Enhancement of CD70-specific CAR T treatment by IFN-gamma released from oHSV-1-infected glioblastoma. Cancer Immunol Immunother.

[21] Seyfrid M, Maich WT, Shaikh VM, Tatari N, Upreti D, Piyasena D, Subapanditha M, Savage N, McKenna D, Mikolajewicz N (2022). CD70 as an actionable immunotherapeutic target in recurrent glioblastoma and its microenvironment. J Immunother Cancer.

[22] Panowski SH, Srinivasan S, Tan N, Tacheva-Grigorova SK, Smith B, Mak YSL, Ning H, Villanueva J, Wijewarnasuriya D, Lang S (2022). Preclinical development and evaluation of allogeneic car T cells targeting CD70 for the treatment of renal cell carcinoma. Cancer Res.

[23] Sauer T, Parikh K, Sharma S, Omer B, Sedloev D, Chen Q, Angenendt L, Schliemann C, Schmitt M, Muller-Tidow C (2021). CD70-specific CAR T cells have potent activity against acute myeloid leukemia without HSC toxicity. Blood.

[24] Razavi A, Keshavarz-Fathi M, Pawelek J, Rezaei N (2021). Chimeric antigen receptor T-cell therapy for melanoma. Expert Rev Clin Immunol.

[25] Ji F, Zhang F, Zhang M, Long K, Xia M, Lu F, Li E, Chen J, Li J, Chen Z (2021). Targeting the DNA damage response enhances CD70 CAR-T cell therapy for renal carcinoma by activating the cGAS-STING pathway. J Hematol Oncol.

[26] Deng W, Chen P, Lei W, Xu Y, Xu N, Pu JJ, Liang A, Qian W (2021). CD70-targeting CAR-T cells have potential activity against CD19-negative B-cell Lymphoma. Cancer Commun.

[27] Park YP, Jin L, Bennett KB, Wang D, Fredenburg KM, Tseng JE, Chang LJ, Huang J, Chan EKL (2018). CD70 as a target for chimeric antigen receptor T cells in head and neck squamous cell carcinoma. Oral Oncol.

[28] Jin L, Ge H, Long Y, Yang C, Chang YE, Mu L, Sayour EJ, De Leon G, Wang QJ, Yang JC (2018). CD70, a novel target of CAR T-cell therapy for gliomas. Neuro Oncol.

[29] Rossotti MA, Belanger K, Henry KA, Tanha J (2022). Immunogenicity and humanization of single-domain antibodies. FEBS J.

[30] Sun S, Ding Z, Yang X, Zhao X, Zhao M, Gao L, Chen Q, Xie S, Liu A, Yin S (2021). Nanobody: a small antibody with big implications for tumor therapeutic strategy. Int J Nanomedicine.

[31] De Munter S, Van Parys A, Bral L, Ingels J, Goetgeluk G, Bonte S, Pille M, Billiet L, Weening K, Verhee A (2020). Rapid and effective generation of nanobody based CARs using PCR and gibson assembly. Int J Mol Sci.

[32] Silence K, Dreier T, Moshir M, Ulrichts P, Gabriels SM, Saunders M, Wajant H, Brouckaert P, Huyghe L, Van Hauwermeiren T (2014). ARGX-110, a highly potent antibody targeting CD70, eliminates tumors via both enhanced ADCC and immune checkpoint blockade. MAbs.

[33] Huang H, Peng T, Hu H, Liu X, Chen Y: Anti-CD70 nanoantibody and use thereof. PCT/CN2022/098843 2022.

[34] Liu Y, An L, Huang R, Xiong J, Yang H, Wang X, Zhang X (2022). Strategies to enhance CAR-T persistence. Biomark Res.

[35] Aspuria PJ, Vivona S, Bauer M, Semana M, Ratti N, McCauley S, Riener R, de Waal Malefyt R, Rokkam D, Emmerich J (2021). An orthogonal IL-2 and IL-2Rbeta system drives persistence and activation of CAR T cells and clearance of bulky lymphoma. Sci Transl Med.

[36] Pal S, Tran B, Haanen J, Hurwitz M, Sacher A, Agarwal N, Tannir N, Budde E, Harrison S, Klobuch S, et al: CTX130 allogeneic CRISPR-Cas9–engineered chimeric antigen receptor (CAR) T cells in patients with advanced clear cell renal cell carcinoma: Results from the phase 1 COBALT-RCC study. Journal for ImmunoTherapy of Cancer 2022;10(Suppl 2):A584-A584.

[37] Maude SL, Laetsch TW, Buechner J, Rives S, Boyer M, Bittencourt H, Bader P, Verneris MR, Stefanski HE, Myers GD (2018). Tisagenlecleucel in children and young adults with B-Cell lymphoblastic leukemia. N Engl J Med.

[38] Neelapu SS, Locke FL, Bartlett NL, Lekakis LJ, Miklos DB, Jacobson CA, Braunschweig I, Oluwole OO, Siddiqi T, Lin Y (2017). Axicabtagene ciloleucel CAR T-cell therapy in refractory large B-cell lymphoma. N Engl J Med.

[39] Schuster SJ, Bishop MR, Tam CS, Waller EK, Borchmann P, McGuirk JP, Jager U, Jaglowski S, Andreadis C, Westin JR (2019). Tisagenlecleucel in adult relapsed or refractory diffuse large B-cell lymphoma. N Engl J Med.

[40] Wang M, Munoz J, Goy A, Locke FL, Jacobson CA, Hill BT, Timmerman JM, Holmes H, Jaglowski S, Flinn IW (2020). KTE-X19 CAR T-cell therapy in relapsed or refractory mantle-cell lymphoma. N Engl J Med.

[41] Ramos CA, Grover NS, Beaven AW, Lulla PD, Wu MF, Ivanova A, Wang T, Shea TC, Rooney CM, Dittus C (2020). Anti-CD30 CAR-T cell therapy in relapsed and refractory hodgkin lymphoma. J Clin Oncol.

[42] Raje N, Berdeja J, Lin Y, Siegel D, Jagannath S, Madduri D, Liedtke M, Rosenblatt J, Maus MV, Turka A (2019). Anti-BCMA CAR T-cell therapy bb2121 in relapsed or refractory multiple myeloma. N Engl J Med.

[43] Shah NN, Highfill SL, Shalabi H, Yates B, Jin J, Wolters PL, Ombrello A, Steinberg SM, Martin S, Delbrook C (2020). CD4/CD8 T-cell selection affects chimeric antigen receptor (CAR) T-cell potency and toxicity: updated results from a phase I anti-CD22 CAR T-cell trial. J Clin Oncol.

[44] Till BG, Jensen MC, Wang J, Qian X, Gopal AK, Maloney DG, Lindgren CG, Lin Y, Pagel JM, Budde LE (2012). CD20-specific adoptive immunotherapy for lymphoma using a chimeric antigen receptor with both CD28 and 4–1BB domains: pilot clinical trial results. Blood.

[45] Martino M, Alati C, Canale FA, Musuraca G, Martinelli G, Cerchione C (2021). A review of clinical outcomes of CAR T-cell therapies for B-acute lymphoblastic leukemia. Int J Mol Sci.

[46] Depil S, Duchateau P, Grupp SA, Mufti G, Poirot L (2020). 'Off-the-shelf' allogeneic CAR T cells: development and challenges. Nat Rev Drug Discov.

