# Supplementary figures and images for: The development of chimeric antigen receptor T-cells against CD70 for renal cell carcinoma treatment
Source: J Transl Med. 2024 Apr 18;22:368. doi: 10.1186/s12967-024-05101-1 (PMC11025280; doi:10.1186/s12967-024-05101-1)

## Slide 1
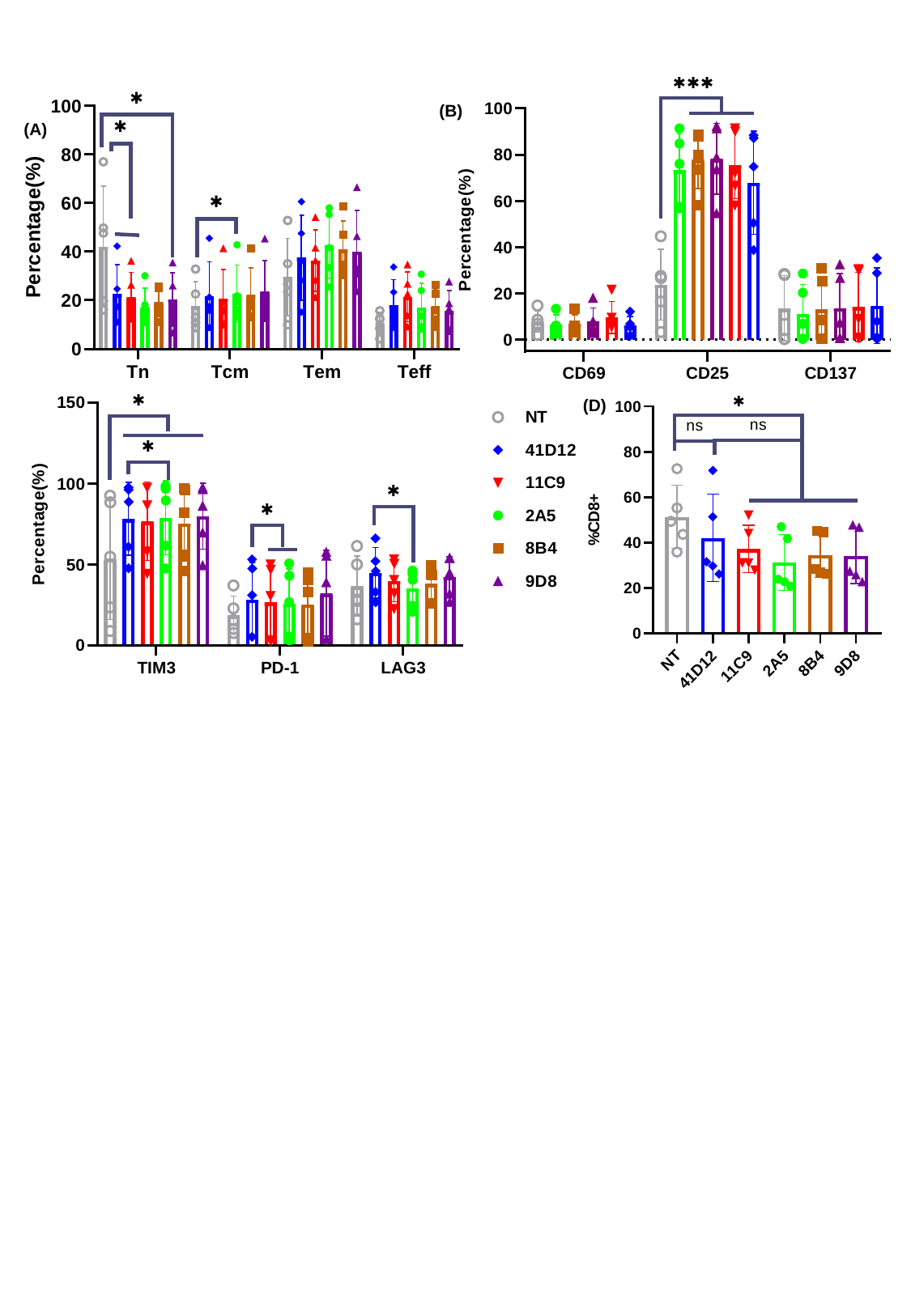

(B)
(A)
(D)

Supplement: Supplementary file 1 — Additional file 1: Figure S1. The Characteristic of CD70 CAR T cells. (A) Phenotypical characterization of CD70 CAR T cells by flow cytometry. differentiated phenotype was determined by the expression of CD45RO and CD62L in 5 different donors. *P<05, by paired Student t test. (B) The activated biomarker expression of CD70 CAR T cells by flow cytometry. Activation was measured by the expression of CD69, CD25, CD137 in 5 different donors ***P<0.001, by paired Student t test. (C). The exhausted marker expression of CD70 CAR T cells by flow cytometry. exhaustion was measured by the expression of Tim3, PD-1 and Lag3 in 5 different donors *P<05, by paired Student t test. (D) The percentage of CD8+ cells in CD3+ cells of CD 70 CAR T cells by flow cytometry. *P<05; ns, not significant, by paired Student t test. [file 12967_2024_5101_MOESM1_ESM.pptx]
