# Supplementary material for: The development of chimeric antigen receptor T-cells against CD70 for renal cell carcinoma treatment
Source: J Transl Med. 2024 Apr 18;22:368. doi: 10.1186/s12967-024-05101-1 (PMC11025280; doi:10.1186/s12967-024-05101-1)
Supplement: Supplementary file 2 — Additional file 2: Figure S2. CD70 expression of human tumor cell lines and T cells in PBMC (A) Analysis of CD70 expression in T cells of a healthy donor’s PBMC by flow cytometry. (B) Analysis of CD70 expression in ACHN wild-type, ACHN-CD70 knockout, k562s wild-type and k562s with CD70 overexpression by flow cytometry. (C)Median fluorescence intensity (MFI) quotients of CD70 versus the respective isotypes on tumor cell lines. [file 12967_2024_5101_MOESM2_ESM.pptx]

## Slide 1
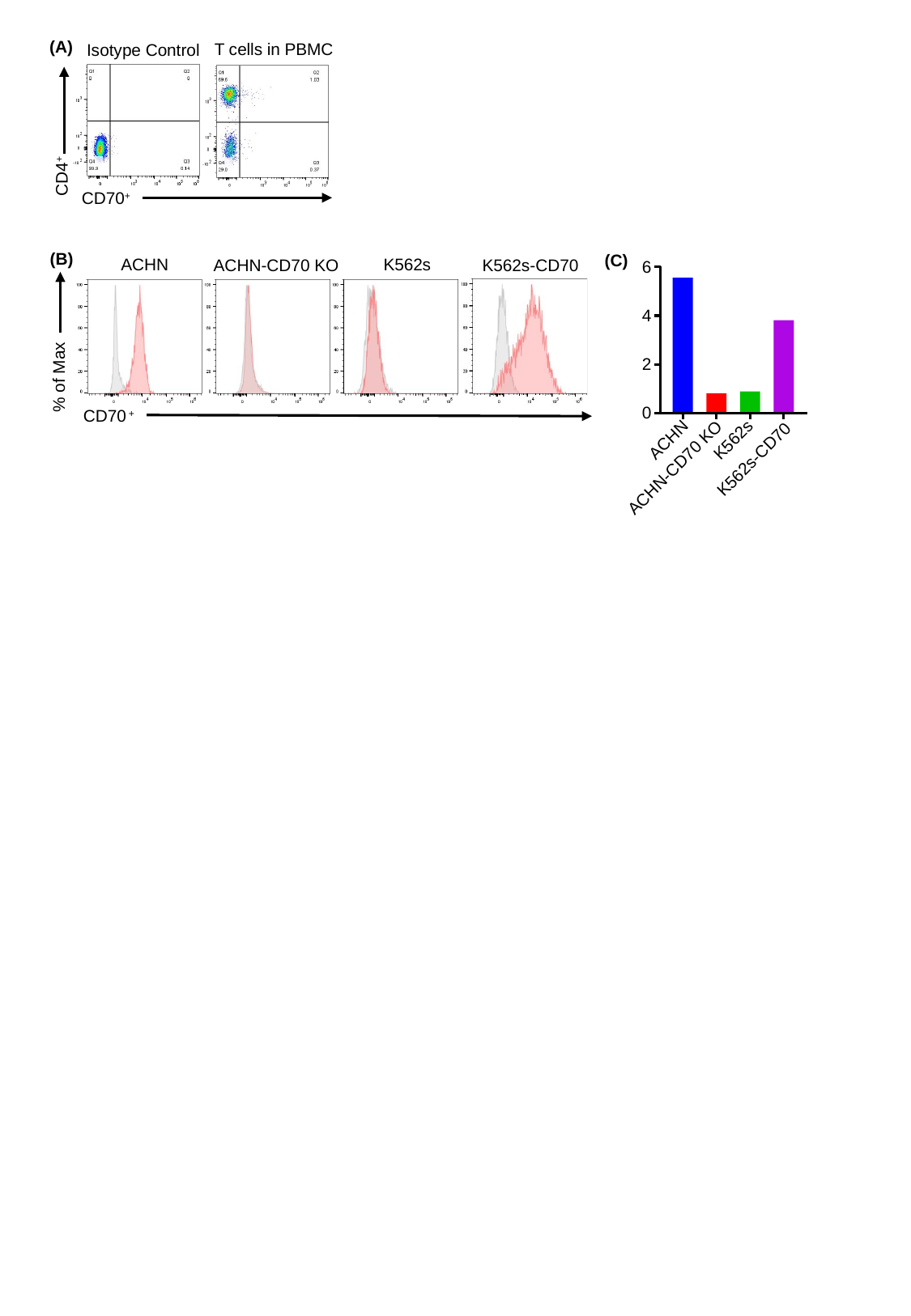

(A)
T cells in PBMC
Isotype Control
CD4+
CD70+
(B)
ACHN
K562s
K562s-CD70
ACHN-CD70 KO
% of Max
CD70 +
(C)
6
4
2
0
ACHN
K562s
K562s-CD70
ACHN-CD70 KO
